# Arterio-venous metabolomics exploration reveals major changes across liver and intestine in the obese Yucatan minipig

**DOI:** 10.1038/s41598-019-48997-2

**Published:** 2019-08-29

**Authors:** Nathalie Poupin, Marie Tremblay-Franco, Aurélien Amiel, Cécile Canlet, Didier Rémond, Laurent Debrauwer, Dominique Dardevet, Ines Thiele, Maike K. Aurich, Fabien Jourdan, Isabelle Savary-Auzeloux, Sergio Polakof

**Affiliations:** 1 Université Clermont Auvergne, INRA, Unité de Nutrition Humaine, UMR1019, Clermont-Ferrand, France; 2 Toxalim (Research Centre in Food Toxicology), Université de Toulouse, INRA, ENVT, INP-Purpan, UPS, Toulouse, France; 3 Luxembourg Centre for Systems Biomedicine, University of Luxembourg, Campus Belval, Esch-sur-Alzette, Luxembourg; 4 Axiom platform, MetaToul-MetaboHUB, National Infrastructure for Metabolomics and Fluxomics, Toulouse, France; 5 School of Medicine, National University of Ireland, University Road, Galway, Ireland; 6 Discipline of Microbiology, School of Natural Sciences, National University of Ireland, University Road, Galway, Ireland

**Keywords:** Metabolomics, Obesity, Homeostasis

## Abstract

Blood circulation mainly aims at distributing the nutrients required for tissue metabolism and collecting safely the by-products of all tissues to be further metabolized or eliminated. The simultaneous study of arterial (A) and venous (V) specific metabolites therefore has appeared to be a more relevant approach to understand and study the metabolism of a given organ. We propose to implement this approach by applying a metabolomics (NMR) strategy on paired AV blood across the intestine and liver on high fat/high sugar (HFHS)-fed minipigs. Our objective was to unravel kinetically and sequentially the metabolic adaptations to early obesity/insulin resistance onset specifically on these two tissues. After two months of HFHS feeding our study of AV ratios of the metabolome highlighted three major features. First, the hepatic metabolism switched from carbohydrate to lipid utilization. Second, the energy demand of the intestine increased, resulting in an enhanced uptake of glutamine, glutamate, and the recruitment of novel energy substrates (choline and creatine). Third, the uptake of methionine and threonine was considered to be driven by an increased intestine turnover to cope with the new high-density diet. Finally, the unique combination of experimental data and modelling predictions suggested that HFHS feeding was associated with changes in tryptophan metabolism and fatty acid β-oxidation, which may play an important role in lipid hepatic accumulation and insulin sensitivity.

## Introduction

The main function of blood circulation is to provide and distribute the nutrients required for tissue metabolism and to collect the by-products that need to be removed from that organ and excreted safely. The modifications of the metabolites in the arterial and venous circulation allow to integrate the whole-body metabolism and therefore to maintain homeostasis. Many molecules are transported in the blood, including nutrients (amino acids, lipids, and carbohydrates), electrolytes and messengers (hormones, peptides, miRNA…). The composition of the blood passing through a tissue is therefore able to reflect its metabolic function: substances taken up by the tissue on a net basis will be in higher concentration in the arterial inflow than in the venous outflow, and vice et versa for released products. Then, for many of these substances, the circulatory system is not just a way to go in and out of an organ, but is also a complex system of cross-talk allowing a whole body integrative metabolism^1^.

Metabolomics has proved to be a valuable tool to further understand metabolic processes^2^. For instance, we and other have shown that untargeted metabolomics was particularly adapted to study the time-course adaptation of metabolism^3–7^. However, so far, the majority of the untargeted studies has focused on biomarkers discovery by profiling blood or urine samples^8^, which provides an interesting static snapshot of the whole body metabolism but offers limited mechanistic information. Indeed, little can be drawn from urine metabolomics about the metabolic activity of a particular organ. Concerning blood, venous samples, which are easily accessible and widely used in clinical diagnosis, likely reflect the metabolism of a particularly-drained organ. On the contrary, arterial blood (exceptionally used in clinical diagnosis) is able to provide a picture of available molecules for the whole body metabolism as it represents the phenotype of the fluid prior to being processed by any particular organ^9^. The simultaneous study of both venous and arterial samples seems therefore a particularly relevant approach to better understand the metabolism a given organ. Assessment of respiratory gases, glucose and amino acids (AA) have been traditionally explored using the arterio-venous (AV) approach since 1930’s in order to perform fundamental metabolic studies at different levels (brain, splanchnic area, kidney, forearm…). However, such studies remained focused on a very limited and targeted set of metabolites or nutrients. Therefore, the global profile of metabolic utilization and exchange of metabolites and nutrients by tissues remains poorly known. In order to obtain an extended view of the organ metabolism beyond biomarker discovery by metabolomics, the assessment of a large number of metabolites in arterial and venous blood is needed. We therefore performed a global profiling of paired arterial and venous (portal and hepatic veins) blood, focusing on the splanchnic area (portal vein-drained viscera and the liver), as the central metabolic crossroad and link between absorption and intermediary metabolism. Samples were collected using a time-course protocol in a minipig model of obesity and insulin resistance (overnutrition with a high fat-high sucrose diet, HFHS)^6,10^ in order to challenge the metabolism with an overload of energy and nutrients. Blood was simultaneously drained at the fasting state before and after 7, 14, 30 and 60 days of a HFHS diet and plasma samples were analysed by NMR-based metabolomics platform. The metabolites showing significant changes between venous and arterial samples were discussed in the context of the biochemical knowledge of these organs and based on the concept of arteriovenous metabostasis, as recently developed by Ivanisevic *et al*. for the study of the skeletal muscle metabolism^11^.

One challenge when interpreting metabolomics data is to explore the direct physiological roles of metabolites and their involvement in organs’ metabolic networks. Metabolic networks can provide a mechanistic context for understanding metabolic profiles and explore the metabolic pathways altered during the onset of obesity. In this study, we therefore integrated the AV metabolic profiles with Genome-Scale Metabolic Network Models (GSMNM) to model the global metabolism of liver and intestine. GSMNMs gather, in an organized and mathematical format, all the biochemical reactions (and associated metabolites) that can occur in a given organism^12^. We used one of the most consolidated reconstruction of the human metabolic network, Recon2.04^13^. It includes more than 7700 reactions and 2600 metabolites, capturing all the reactions that can possibly occur in any human tissue or cell. Constraint-based modelling (CBM) methods then allow to predict the metabolic state of a tissue, by setting bounds (lower and upper constraints) to some specific reactions based on available experimental data^14^. In this study, constraints were set on exchange reaction fluxes in the models to enforce uptake or release of metabolites according to the consumption and production profiles. This modelling strategy allows contextualizing the generic model and generating time- and tissue-specific constrained models, which can specifically account for the functional metabolic network in these conditions.

## Materials and Methods

### Animals and experimental procedure

The study involved five female adult Yucatan mini-pigs (31.5 ± 1.4 kg). Three weeks before the experimentation, mini-pigs were surgically fitted with a catheter in the abdominal artery aorta (Art), the portal vein (PV) and the hepatic vein (HV). They were housed in subject pens (1 × 1.5 m) in a ventilated room with controlled temperature (21 °C) and regular light cycle (L12:D12). They were fed once daily with 400 g/d of a concentrate feed containing 17.5% protein, 3.2% fat, 4.3% cellulose, and 5.2% ash (Porcyprima; Sanders Centre Auvergne, Aigueperse, France) and had free access to tap water. All procedures were in accordance with the guidelines formulated by the European Community for the use of experimental animals (L358-86/609/EEC, Council Directive, 1986). The protocol was approved by the Ethical Committee for Animal Experimentation Auvergne, authorization 02090.01.

After the recovery period, minipigs were fed a HFHS diet consisting in a regular pig diet enriched with fat (13% palm oil) and sugar (10% sucrose) (1 kg/day, 15.5 kJ/day) for two months. Animals ingested the whole mixture in no more than 10 minutes. After an overnight fasting, blood was sampled through the three catheters simultaneously on heparinized tubes, before (d0), and 7, 14, 30, and 60 days after HFHS feeding. Blood was centrifuged at 4,500 × g for 10 min, plasma rapidly collected and stored at −80 °C until further analyses. Biochemical and phenotype measures were also performed at the different time points: the related procedures and data have been previously published^10^.

### Plasma metabolomics

200 µL of plasma samples were mixed with 500 µL of phosphate buffer (pH 7.0) prepared in deuterated water, and then centrifuged at 5500 *g* at 4 °C for 15 minutes, and 600 µL of supernatant were transferred to 5 mm NMR tubes. All ^1^H NMR spectra were obtained on a Bruker DRX-600-Avance NMR spectrometer operating at 600.13 MHz for ^1^H resonance frequency using an inverse detection 5 mm ^1^H-^13^C-^15^N cryoprobe attached to a Cryoplatform (the preamplifier unit). The ^1^H NMR spectra were acquired at 300 K using the Carr-Purcell-Meiboom-Gill (CPMG) spin-echo pulse sequence with presaturation, with a total spin-echo delay (2nτ) of 240 ms to attenuate broad signals from proteins and lipoproteins. A total of 256 transients were collected into 32k data points using a spectral width of 20 ppm, a relaxation delay of 2 s and an acquisition time of 1.36 s. Prior to Fourier Transformation, an exponential line broadening function of 0.3 Hz was applied to the FID. All NMR spectra were phased and baseline corrected, then data were reduced using AMIX (version 3.9 Bruker, Rheinstetten, Germany) to integrate 0.01 ppm wide regions corresponding to the δ 8.6–0.7 ppm region. The δ 5.1–4.5 ppm region, which includes the residual water resonance, was excluded. A total of 581 NMR buckets were included in the data matrix. To account for differences in sample concentration, each integrated region was normalized to the total spectral area. For the exchange of metabolites across the organs we calculated the ratios between the artery and the veins: Art/PV for the intestine, and [(Art × 0.2) + (PV × 0.8)]/HV for the liver. An averaged relative contribution of 20% of the hepatic arterial flow (Art) to the total hepatic flow was used as an approximation based on previously reported results^15^. We then expressed these ratios as percentage of metabolisation from steady state, considering steady state as inflow/outflow = 1.

$$ \% \,{{\rm{metabolisation}}}_{({\rm{met}}{\rm{m}},{\rm{indiv}}{\rm{i}},{\rm{day}}{\rm{d}})}=(\frac{{{\rm{Art}}}_{{\rm{met}}{\rm{m}},{\rm{indiv}}{\rm{i}},{\rm{day}}{\rm{d}}}}{{{\rm{PV}}}_{{\rm{met}}{\rm{m}},{\rm{indiv}}{\rm{i}},{\rm{day}}{\rm{d}}}}-1)\times 100,\,{\rm{for}}\,{\rm{intestine}};$$

$$\begin{array}{c} \% \,{{\rm{metabolisation}}}_{({\rm{met}}{\rm{m}},{\rm{indiv}}{\rm{i}},{\rm{day}}{\rm{d}})}\\ \,=(\tfrac{{{\rm{Art}}}_{{\rm{met}}{\rm{m}},{\rm{indiv}}{\rm{i}},{\rm{dayd}}}\times 0.2+{{\rm{PV}}}_{{\rm{met}}{\rm{m}},{\rm{indiv}}{\rm{i}},{\rm{day}}{\rm{d}}}\times 0.8}{{{\rm{PV}}}_{{\rm{met}}{\rm{m}},{\rm{indiv}}{\rm{i}},{\rm{day}}{\rm{d}}}}-1)\times 100,\,{\rm{for}}\,{\rm{liver}};\end{array}$$

where Art, PV and HV are the NMR integration area of each metabolite measured in plasma samples from Art, PV and HV respectively. For each metabolite, a signal without overlapping has been chosen for integration ratio calculation. %metabolisation for all measured metabolites are presented in Suppl. Fig. 1.

### Statistical analyses

Multivariate analyses were used to study the effect of high fat diet along time course on the metabolome. Principal components analysis (PCA) was first performed to reveal intrinsic clusters and detect eventual outliers. Partial least squares-discriminant analysis (PLS-DA) was then used to model the relationship between group and spectral data. PLS-DA is similar to PCA but uses discriminant variables that correlate to class membership. Before analysis, orthogonal signal correction (OSC) filtering was used to remove variability not linked to the studied conditions (physiological, experimental or instrumental variation). Filtered data were mean-centered and Pareto scaled. For all the figures, Hotelling’s T2 statistics were used to construct 95% confidence ellipses. The R2Y parameter represents the explained variance. Seven-fold cross validation was used to determine the number of latent variables to include in the PLS-DA model and to estimate the predictive ability (Q^2^ parameter) of the adjusted model. In addition, the robustness and validity of the PLS-DA models were calculated using a permutation test (number of permutations = 200). Significant NMR variables were identified using 1D and 2D NMR spectra. Discriminated time points (i.e. with different metabolic profiles) d0, d7 and d60 were selected based on the multivariate statistical analyses. For these three time points, comparisons between venous and arterial blood across the organs were performed using t-paired Student tests and the time effect was assessed using a repeated-measures one-way ANOVA test. SIMCA-P software (V13, Umetrics AB, Umea, Sweden) was used to perform the multivariate analyses. SigmaPlot v12.3 (Systat Software, San Jose, CA) was used to perform the univariate analyses.

#### Metabolic network modelling

For each time point (d0, d7 and d60) and for each of the metabolites measured experimentally, paired t-tests between Art and PV and between HV and (Art × 0.2 + PV × 0.8) were performed, to identify metabolites significantly released or taken up by the gut or the liver. Metabolites with p-value < 0.05 and with a metabolisation % higher than 5% were considered as taken up by the tissue, whereas metabolites with p-value < 0.05 and with a metabolisation % lower than −5% were considered as released. For the metabolisation % between −5 and +5% the metabolites were considered as not exchanged or used by the tissue. The remaining metabolites, with a % metabolisation lower than −5% or higher than +5% but with a p-value > 0.05, were considered as not significantly exchanged and therefore were not constrained in the modelling.

We integrated these extratissular exchange data into the GSMNM Recon2^16^ (version 2.04, downloaded from http://vmh.uni.lu/#downloadview)^17^ to simulate metabolic fluxes through intratissular reactions and predict the changes in gut and liver metabolic state during HFHS feeding. The Recon2 model contains 7440 reactions and 2626 metabolites, including 642 exchange reactions and associated metabolites which can potentially be taken up or secreted from the model. All these exchange reactions are initially unbounded by default (with lower and upper bounds set to −500 and +500 respectively), allowing uptake and secretion of all these metabolites. By definition of the exchange reactions in the model, a negative flux through an exchange reaction corresponds to an uptake of metabolite (incoming flux into the model), whereas conversely a positive flux corresponds to a release of metabolite (outgoing flux out of the model). Acquired metabolomics data were used to set specific constraints on these exchange reactions considering the metabolites evidenced as taken up, secreted and not exchanged from metabolomics data at each time. For released metabolites, the corresponding exchange reactions were constrained to enforce minimal release (*i*.*e*., minimal positive flux through exchange reaction), by setting the upper bound (ub) to the flux equivalent to the limit of detection (LOD) value (Suppl. Fig. 2). Similarly, for metabolites taken up, corresponding exchange reactions were constrained to enforce minimal negative flux through exchange reaction (lower bound (lb) set flux equivalent to –LOD). For metabolites considered as not exchanged (with % metabolisation within −5% and +5%), the corresponding exchange reactions were constrained to have a limited absolute flux value, lower than the LOD. Of note, contrary to not exchanged metabolites, no constraints were set for metabolites whose changes in level between inflow and outflow were not found to be significant (both uptake and release were *a priori* allowed). The concentration was defined as LOD in ^1^H NMR spectroscopy when the signal-to-noise ratio (S/N) of signals reached 3:1. LOD was estimated at 25 μM in NMR tube for signals corresponding to 3 protons, i.e. 87.5 uM in plasma samples. LOD was estimated at 75 μM in NMR tube for signals corresponding to one proton, i.e. 260 μM in plasma samples. LOD, were converted to equivalent fluxes using an estimated blood flow value of 100 ml/g liver/h^18^. Constraints were set with a 20% margin from the LOD. Three of the experimentally exchanged metabolites (betaine, glycerophosphocholine and ethanolamine) could theoretically not be exchanged in the Recon2 model as they were not associated to any exchange reactions: exchange reactions and extracellular transport reactions were added for these metabolites to allow their import and export. All exchange reactions not constrained from experimental data were set to secretion only (lb = 0), except for a list of metabolites which are considered as being always present in plasma and for which uptake from plasma was allowed in the model. A constrained model was generated for each time point and each tissue, therefore leading to 6 time- and tissue-specific models: SI-d0, SI-d7, SI-d60, Liver-d0, Liver-d7 and Liver-d60. The list of constraints defined for each model in given in Suppl. Table 1.

The coefficients of the biomass reactions were adapted to better fit with the composition of an hepatic cell using data from the literature (Suppl. Table 2)^13,19^ and to account only for tissue maintenance (deoxyribonucleotides were excluded). Coefficients were also rescaled to get fluxes in µmol/g tissue/h instead of mmol/gDW/h (using an estimate of 3.8 for the liver Wet Weight (WW)/Dry Weight (DW) ratio)^20^. The lower bound for the biomass reaction was constrained in order to get a minimal protein fractional synthesis rate of 5%.h^−1^ ^21^ and the albumin production and release was allowed in the 3 liver-specific models.

The possible range of flux values through each reaction (minimal and maximal possible fluxes) was computed using Flux Variability Analysis (FVA)^22,23^. FVA results are provided in Suppl. Table 3. To compare the simulated flux ranges between pairs of time points, we calculated the Jaccard distance of each reaction as the ratio of the intersection to the union of the flux ranges at 2 different time points. For instance, for reaction i, the Jaccard distance between simulated flux ranges at d0 ([min_d0_; max_d0_]) and d7 ([min_d7_; max_d7_]) is computed as follow:

$$\begin{array}{rcl}{{\rm{J}}}_{{\rm{i}},{\rm{d}}0-{\rm{d}}7} & = & \frac{\mathrm{intersection}\,({[{\rm{\min }};{\rm{\max }}]}_{{\rm{d}}0},{[{\rm{\min }};{\rm{\max }}]}_{{\rm{d}}7})\,}{\mathrm{union}\,({[{\rm{\min }};{\rm{\max }}]}_{{\rm{d}}0},{[{\rm{\min }};{\rm{\max }}]}_{{\rm{d}}7})}\\  & = & \frac{\min ({{\rm{\max }}}_{{\rm{d}}0};{{\rm{\max }}}_{{\rm{d}}7})-\,{\rm{\max }}({{\rm{\min }}}_{{\rm{d}}0};{{\rm{\min }}}_{{\rm{d}}7})}{\max ({{\rm{\max }}}_{{\rm{d}}0};{{\rm{\max }}}_{{\rm{d}}7})-\,{\rm{\min }}({{\rm{\min }}}_{{\rm{d}}0};{{\rm{\min }}}_{{\rm{d}}7})}\end{array}$$

This represents the overlap between the possible flux values at d0 and d7: J distance of 0 indicates that the 2 flux intervals do not overlap at all and therefore that the reaction i has distinct flux values at d0 and d7, whereas a J distance of 1 (100%) indicates that there is a complete overlap between possible flux values at d0 and d7.

For each pairwise time comparison and each tissue, we performed a pathway enrichment analysis on the reactions with J distance lower than 1. This aims to assess whether the reactions with modulated flux values between 2 time points are significantly over-represented in a metabolic pathway. Pathway enrichment statistics were performed using one-tailed exact Fisher test, with a Bonferroni correction for multiple tests^24^, using the metabolic pathways defined in Recon2.

## Results

After two months of HFHS feeding minipigs developed an obesity-like phenotype, with a significant increase in body weight (from 31.5 ± 1.4 kg to 44.7 ± 1.7 kg), most likely as the consequence of fat deposition at the visceral and subcutaneous adipose tissue^6^. More details about the biochemical and clinical phenotyping have been previously published^10,25^.

**Figure 1:**
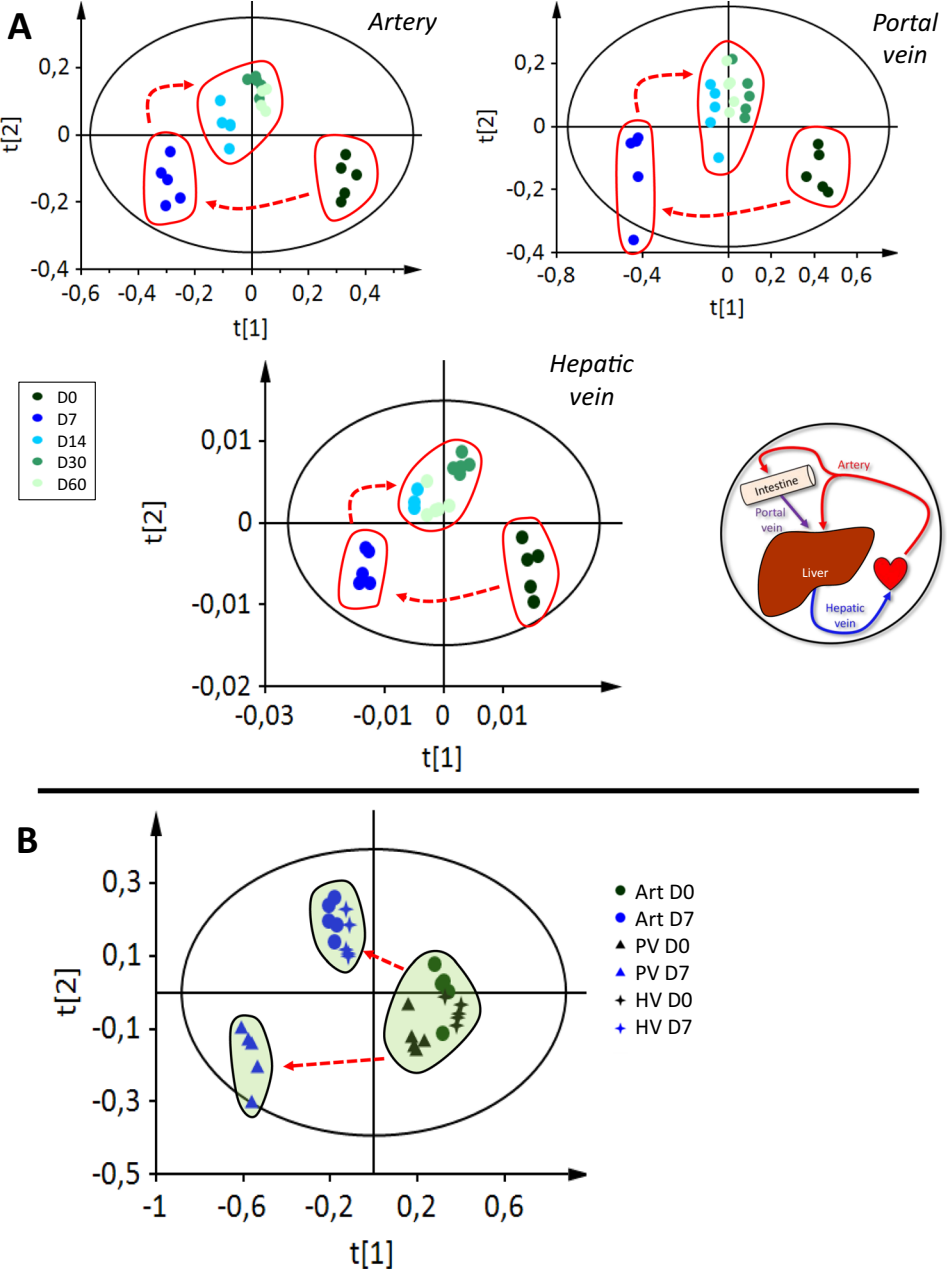
(**A**) Scores plot of partial least squares discriminant analysis model (n = 5) after OSC filter for five classes: d0, d7, d14, d30 and d60 of HFHS feeding. For the artery, R² = 64.6%, Q² = 0.51; for the portal vein, R² = 44.4%, Q² = 0.31; for the hepatic vein, R² = 64.3%, Q² = 0.47. (**B**) Scores plot of partial least squares discriminant analysis model (n = 5) after OSC filter for six classes: artery (Art), portal vein (PV) and hepatic vein (HV) and d0 and d7 of HFHS feeding. R² = 37.4%, Q² = 0.32, two latent variables.

The trajectory of the metabolome obtained from different sampling sites, *i*.*e*., PV, Art and HV is shown in Fig. 1. Clustering of observations is very similar between the vessels. Three clusters depending on the duration of HFHS diet feeding were observed: d0, d7, d14 and d60. After d14, the phenotype was no longer discriminated from those of d30 or d60. In all cases, the model was valid and robust, including three latent variables, R² = 64.4% Q² = 0.51 for the Art; two latent variables, R² = 41.4% and Q² = 0.31 for the PV; and three latent variables, R² = 64.3% and Q² = 0.47 for the HV. Because d14 and d30 were not metabolically distinct from d60, only d0, d7 and d60 were selected for further analyses.

Based on the results presented in the Fig. 1A, we decided to analyse separately the metabolic profiles of all vessels between d0 and d7, where the major shift occurred. We obtained a valid and robust model (R² = 37.4% and Q² = 0.32) that did not discriminate the metabolomes from the different vessel at d0 (Fig. 1B). However, at d7 the metabolome of the PV was clearly discriminated from that of the HV and the Art. All the metabolomes at d0 were also discriminated from those at d7.

**Figure 2:**
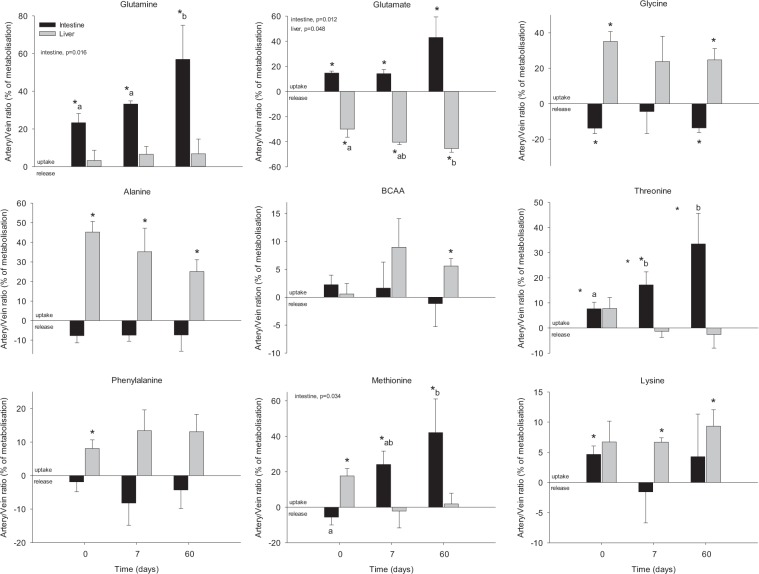
Arteriovenous fold-change (A/V) across the splanchnic area (liver and portal drained viscera) of amino acids on five Yucatan minipigs submitted to a HFHS diet during two months. Data is presented in % of metabolisation (mean + sem): positive values = taken up by the organ; negative values = released by the organ. Based on the results obtained in the Fig. 1, only day 0, 7 and 60 are shown. Data were analysed using a repeated-measures one-way ANOVA test (p-value for time is shown when < 0.05). *Significantly different from 0 (equilibrium) (P < 0.05). Different letter indicate significant differences among the days, p < 0.05.

**Figure 3:**
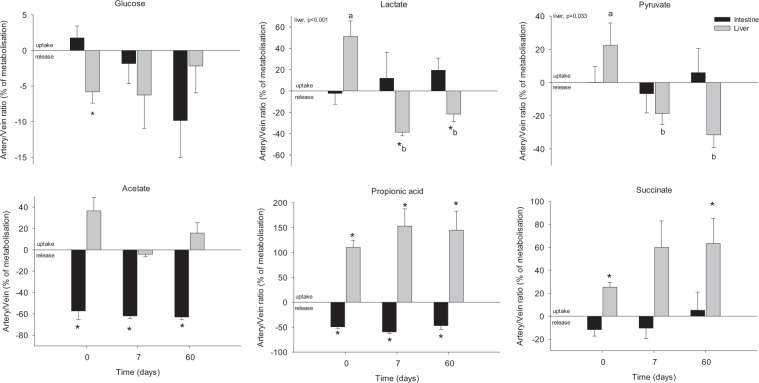
Arteriovenous fold-change (A/V) across the splanchnic area (liver and intestine) of energy-related metabolites on five Yucatan minipigs submitted to a HFHS diet during two months. Data is presented in % of metabolisation (mean + sem): positive values = taken up by the organ; negative values = released by the organ. Based on the results obtained in the Fig. 1, only day 0, 7 and 60 are shown. Data were analysed using a repeated-measures one-way ANOVA test (p-value for time is shown when < 0.05). *Significantly different from 0 (equilibrium) (P < 0.05). Different letter indicate significant differences among the days, p < 0.05.

The global profiling of inflow and outflow blood samples measured simultaneously using NMR metabolomics approach highlighted the significant changes in arterial *vs*. venous plasma during circulation across the gut and liver of HFHS minipigs. Based on the PLS-DA results those metabolites with significant different levels in venous *vs*. arterial plasma at d0, d7 or d60 are represented in Figs 2 and 3 (as % of metabolisation from the steady state). Direction of the % of metabolisation change implies either a positive AV balance, reflecting metabolite uptake by the organ, or a negative arteriovenous balance, reflecting metabolite release by the organ^11^. Among the significant changing metabolites, several AA were identified displaying consistently shifted ratios in time across the gut: higher arterial vs. venous levels were observed for glutamine (20–60% of metabolisation), glutamate (5–40% of metabolisation), threonine (20–30% of metabolisation, except at d0), and methionine (20–40% of metabolisation, except at d0), whereas increased venous levels were found for glycine (no more than −20% of metabolisation at d0 and 60). Several of these AA showed also modified levels between the inflow (Art and PV) and outflow (HV) across the liver. Opposite trend compared to gut was observed for glutamate and glycine, with a release profile for glutamate (−35% metabolisation) and an uptake profile for glycine (20–40% metabolisation). Higher levels of alanine (20–45% of metabolisation), methionine (18% of metabolisation at d0, but no metabolisation at d7 and d60), lysine (10% of metabolisation at d7 and 60), and phenylalanine (8% of metabolisation at d0) were found in the inflow plasma when compared to the outflow levels across liver.

Several metabolites directly involved in the energy flow through metabolism were also found to significantly differ in arterial *vs*. venous plasma across liver, whereas no significant differences were evidenced between inflow and outflow across the gut (Fig. 3). Among them, several metabolites participating to the hepatic glucose metabolism were identified, including glucose, lactate, pyruvate and succinate. Glucose displayed higher concentration in outflow (hepatic vein) blood than in the inflow (arterial and portal) blood at d0 (averaging −6% of metabolisation), whereas this differential release was no longer observed on d7 and d60. Inversely, lactate and pyruvate appeared to be released (with a −20 to −40% metabolisation) at later times only (d7 and d60). Succinate also showed a differential utilisation across the liver, but with a clear uptake pattern, increasing from a 20% metabolisation at d0, up to 60% at d60. Two metabolites produced by the gut microbiota, namely acetate and propionate, were also identified. While for propionate the profile clearly shows an inverse pattern between gut and liver, with a positive balance (up to 100% metabolisation between d7-60) at the liver level and a negative balance (−50%) for the gut, the acetate results are less clear, with a significant negative balance evidenced only across the gut (averaging −50% of metabolisation) irrespectively of the day.

**Figure 4:**
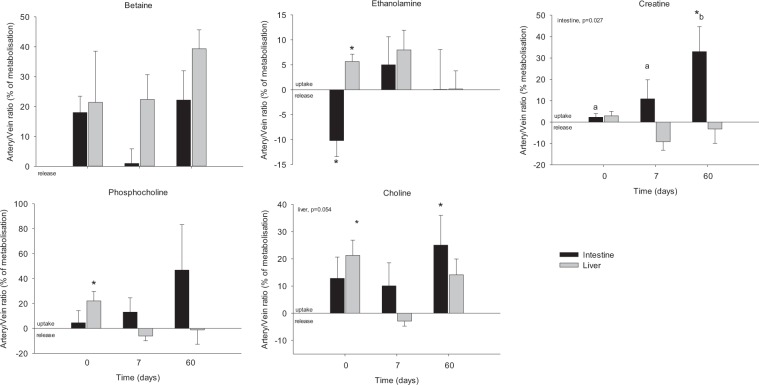
Arteriovenous fold-change (A/V) across the splanchnic area (liver and intestine) of metabolites on five Yucatan minipigs submitted to a HFHS diet during two months. Data is presented in % of metabolisation (mean + sem): positive values = taken up by the organ; negative values = released by the organ. Based on the results obtained in the Fig. 1, only day 0, 7 and 60 are shown. Data were analysed using a repeated-measures one-way ANOVA test (p-value for time is shown when < 0.05). *Significantly different from 0 (equilibrium) (P < 0.05). Different letter indicate significant differences among the days, p < 0.05.

Other small metabolites showing significant differences between the venous and arterial blood samples were further identified, including betaine, ethanolamine, creatine, phosphocholine and choline (Fig. 4). While creatine and choline showed a clear pattern of uptake by the gut particularly significant at d60 (about 30% of metabolisation), betaine showed a positive metabolisation across the liver at d7 and 60 (averaging 30%), and across the gut (about 20% of metabolisation) at d0. Finally, we recorded a positive metabolisation across the liver at d0 for phosphocholine (20%) and ethanolamine (5%), along with a negative metabolisation of the latter across the gut.

**Figure 5:**
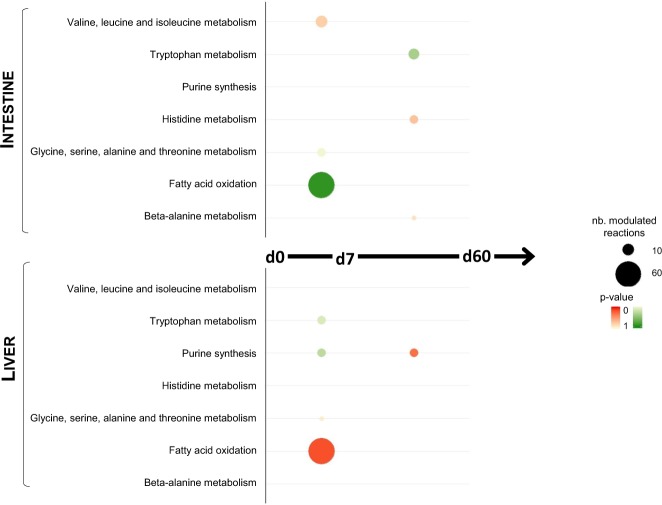
Predicted modulated metabolic pathways. Pathway enrichment analysis showing Recon 2 pathways that are contain a significantly high number of reactions which predicted flux ranges differ between time points (as assessed by a Jaccard distance lower than 1). The size of the circles is proportional to the number of differing reactions. The colour of the circles depends on the p-value (one-tailed exact Fisher test, with Bonferroni correction for multiple tests). Pathways for which reactions tend to have a larger (resp., tighter) flux range at d7 than d0 and at d60 than d7 are coloured in green (resp. red).

Significant observed changes in metabolites levels between inflow and outflow across gut and liver were used to model the changes in gut and liver metabolism at d0, d7 and d60. Based on the uptake and release of metabolites at each time, possible ranges for intratissular fluxes were simulated for all the reactions defined in the human metabolic network Recon2 (Suppl. Table 3). Changes in fluxes were compared between d0 and d7 and between d7 and d60 for gut and liver separately. We predicted that about 100 reactions would have potentially different flux values between d0 and d7, but no more than 20 reactions between d7 and d60 (Suppl. Table 4). These predicted altered reactions between d0 and d7 mostly belong to the fatty acid oxidation pathway (Fig. 5 & Suppl. Table 5). Reactions involved in the tryptophan degradation pathway are also significantly present among the predicted changed reactions between d0 and d7 for liver, but between d7 and d60 for intestine. Also, the purine synthesis pathway included a significant number of predicted modulated reactions for liver (8 reactions), with a trend to increased possible fluxes from d0 to d7 and reduced possible fluxes from d7 to d60.

## Discussion

The paired arteriovenous metabolomics data-driven study presented here provides a comprehensive overview of the metabolite exchange and utilization across the splanchnic area in normal and obese minipigs. While in previous studies single organs have been explored using this approach (muscle in healthy humans^11^ and mammary gland in dairy cows^26^), this is the first time that such strategy is simultaneously applied to two organs (liver and intestine) in a pathophysiological model (obesity). Applying this AV measurement strategy for *in vivo* exploration using the obese minipig model offers two main advantages: first, to solve the problem of the PV access in humans^27^; and second, to be able to perform a follow up on the onset of obesity on the same individuals. We evidenced a similar metabolic trajectory in each blood vessel metabolic profile, with a clear discrimination of three steps: d0, d7 and d14 to d60, which is consistent with our previous study of the urine metabolome trajectory^6^. We therefore focused on the comparison of the metabolites exchanges in healthy state (d0) and in early (d7) and late (d60) obesity stages. The metabolomics data obtained across the intestine and the liver were also analysed by constraint-based mathematical modelling, which allowed to formulate further explanations about the fate of the exchanged metabolites.

### Characterization of the splanchnic metabolism based on the AV differences in healthy minipigs

**Figure 6:**
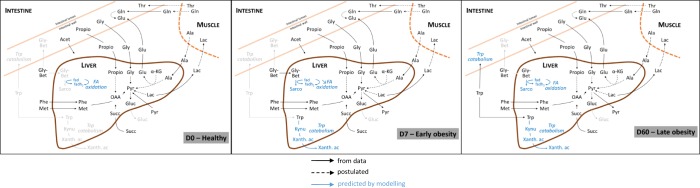
Schematic representation of the main biochemical reactions observed across the splanchnic area (intestine and liver) in HFHS-fed minipigs for 3 different stages in the onset of obesity. Exchange fluxes represented in the figure are based on the inflow to outflow ratios measured by NMR and the modelling results. Acet, acetate; Ala, alanine; Bet, betaine; Gln, glutamine; Gluc, glucose; Glu, glutamate; Gly, glycine; Kynu, kynurenine; Lac, lactate; OAA, oxaloacetate; Phe, phenylalanine; Pyr, pyruvate; sarco, sarcosine; Thr, threonine; Trp, tryptophan; Xanth ac, xanthurenic acid.

At day 0 the liver metabolism shows a classical postabsorptive profile. The major role of the liver during this period is to provide glucose to the peripheral tissues through the *de novo* glucose production (gluconeogenesis)^28^ (Fig. 6A). In our study, we indeed observed a negative A/V balance of glucose across the liver, demonstrating its hepatic production. We also observed that different substrates like lactate, pyruvate or AA^29^ could be delivered from other organs to the liver in order to produce glucose. Several gluconeogenic AA were actively taken up by the liver, including glycine, alanine, phenylalanine and methionine. While glycine seemed to have an intestinal origin, the rest of AA were most likely released from the skeletal muscle. In the particular case of alanine, its release from the muscle participates in the glucose-alanine cycle^30^ and can also explain the glutamate liver release, as the result of alanine transamination. Lactate and pyruvate, also tend to be taken up by the liver, which could contribute to the concomitant glucose output. Given that lactate was not released by the intestine, its origin would be likely the muscle, as previously shown in fasted pigs^30^ and as part of the Cori cycle.

The intestine is a metabolically active organ^31^: during the postabsorptive period, its AA supply comes mainly from the artery and is especially fueled by glutamine^32^ and glutamate^31^, which are used for energy purposes^33^. In our study, these two AA were actively taken up by the intestine, which is in agreement with the extensive glutamine utilization by the pig intestine^31^. The precise origin of glutamine could not be determined in the present study, but it would be likely released from the skeletal muscle^30^, while according to our data glutamate was rather released by the liver.

Finally, both, acetate and propionate showed a positive A/V ratio across the liver and a negative ratio across the intestine, reflecting their intestinal production and their hepatic uptake from the portal circulation. Acetate has been shown to be used as an energy substrate by the liver through its oxidation by the tricarboxylic acid cycle (TCA), but also as a substrate for the synthesis of cholesterol and long-chain fatty acids^34^. Concerning propionate, it is also known that the liver clears the major part of this metabolite from the portal circulation^35^, and that it is mostly used for gluconeogenesis purposes^36^, which is further consistent with our results.

### Effects of HFHS feeding: focus on the first week

From the metabolome trajectory, we noticed that the phenotype of all vessels shifted a d7 (Fig. 1A). We therefore performed at this particular time a blood vessel comparison of the phenotype, evidencing that the metabolome of the PV is clearly discriminated from those of the Art and HV (Fig. 1B). Interestingly the modelling results also support this idea of a larger metabolic shift during this first week of HFHS feeding, with a larger number of reactions having potentially modulated flux ranges between d0 and d7 than between d7 and d60 (Fig. 5).

In contrast to the situation observed at d0, where several metabolites involved in phospholipids metabolism (methionine, choline, phosphocholine and ethanolamine) were actively taken up by the liver, their uptake was blunted after only one week of HFHS feeding (Fig. 6B). A reduced synthesis of phospholipids could be deleterious for the liver, as insufficient phosphocholine supplies will inevitably cause triglyceride accumulation and eventually fatty liver^37^.

After one week of HFHS feeding, we observed a reduced glucose release from the liver, most likely due to a reduced utilization of gluconeogenic substrates, like lactate and AA (methionine, phenylalanine). During the postabsorptive period, gluconeogenesis is predominantly fuelled by lactate^38^, which at d7 was no longer taken up by the liver, but rather released. This is also consistent with a reduction in glucose catabolism, which could further explain the pyruvate accumulation and release^39,40^. Thus, the increased availability of lipids could displace the use of pyruvate as a substrate for oxidative metabolism. Pyruvate will then cumulate and be subsequently diverted into lactate, resulting in both pyruvate and lactate release from the liver. In the same line, we observed that succinate was importantly taken up by the liver. In high substrate availability contexts, such as HFHS feeding, succinate could be released from tissues^41^. Under these conditions, the TCA may increase the H^+^ gradient over the mitochondrial membrane, leading to inhibition of enzymatic steps mediated by complexes within the electron transport chain and eventually, release of succinate into the blood stream^42^. In mice fed on a high fat diet and supplemented with sodium succinate, a reduced lipid accumulation in the liver was observed^43^. We may therefore consider that the early (d7) increased succinate uptake by the liver of the HFHS-fed minipigs could be part of a strategy aiming at reducing hepatic steatosis as the consequence of the deleterious fat and sugar intake. Despite a maintained intestinal release of acetate under HFHS feeding, its uptake by the liver was blunted after d7, which is consistent with the strategy to reduce lipid production from this substrate, by redirecting the intestinal acetate to peripheral organs for further oxidation^44^.

As a whole, in the first week of HFHS feeding, we observed several features suggesting that the minipig metabolism rapidly reprogrammed and that there might be a metabolic shift at the hepatic level toward utilizing the most abundant nutrients (i.e. lipids) while alleviating the potentially associated deleterious effects.

### Longer-term effects of HFHS feeding

Globally the shifts observed at d7 tend to persist at d60 as suggested by the fewer changes in metabolome observed between d7 and d60 (Fig. 6C). The major impact of two months of HFHS feeding on the intestine metabolism can be summarized by an increased energy demand coped by an enhanced metabolism of several metabolites. Glutamine and glutamate are the preferred substrates to fuel the physiological processes stimulated by the HFHS feeding, like epithelium renewal and expansion and cellular turnover^45^. The positive A/V balance of glutamine and glutamate is significantly increased from d0 to d60. Interestingly, the energy demand seems to be complemented by other metabolites poorly used at d0, like creatine, a major metabolite involved in energy metabolism^46^. Creatine could be then preferentially used to rapidly yield energy^47^, which would be consistent with the positive correlation found between the adiposity and creatine levels in HF-fed pigs^48^.

HFHS consumption may induce cellular stress and inflammation at the intestinal level^49^. Some metabolic features observed in this study are consistent with this hypothesis and could be related to biological processes aiming at developing the intestinal functionality. For instance, methionine and threonine were more highly taken up after two months of HFHS consumption. This is consistent with the fact that methionine participates in the epithelial cell turnover as well as in the maintenance of the antioxidant status (by keeping cysteine available) and the cell level of reduced glutathione^50^, while threonine is largely used for mucins synthesis in pig mucosa^51,52^, which is of outmost importance in epithelium protection and innate immune defence.

In parallel, we also reported that glutamate A/V across the liver was further increased at d60, suggesting its larger exportation into the peripheral circulation. Barber *et al*. reported that in cafeteria-fed animals glutamate was released from the liver while urea production was reduced^53^, which is consistent with our previous observations^6^ and could reflect a nitrogen sparing mechanism already observed in other situations^54,55^. We may therefore suggest that these changes would result from a metabolic switch in which AA catabolism would be reduced in detriment of lipids utilization, highly abundant in the HFHS diet, and able to induced a reduction in the urea cycle in both rodents^53,56^ and pigs^10^.

### Splanchnic metabolism in obese HFHS-fed minipigs: insights from mathematical modelling

In order to go deeper into the exploration of the changes in splanchnic metabolism under HFHS feeding, we have submitted the AV metabolomics data to constraint-based mathematical modelling. Our results allowed gaining new insights into the intrahepatic routing of exchanged metabolites experimentally assessed by NMR and mainly predicted changes in three metabolic pathways: fatty acid oxidation, tryptophan metabolism and purine synthesis (Fig. 5).

Our *in silico* simulations predicted that the metabolic fluxes through the tryptophan pathway reactions tend to be increased towards the production of kynurenine and its downstream metabolites, some of them (like 3-hydroxy-kynurenine, 2-Amino-3-carboxymuconate semialdehyde) being major crossroads for the production of kynurenate, xanthunerate or quinolenate^57^. These metabolites were shown to be elevated in obese and insulin resistant subjects, and some of the metabolites participating at these particular pathways are known to reduce insulin production and sensitivity in laboratory animals^58,59^. Further, changes in tryptophan and its related metabolites have been associated with altered indoleamine 2,3-dioxygenase-1 activities, one of the rate-limiting enzyme of tryptophan catabolism and a possible indirect indicator of immune mediated inflammation in obese and overweight conditions^60,61^.

We also predicted that the activity of the purine synthesis pathway would be transitory increased in liver after 7 days of HFHS diet, with a potentially larger flux through a set of reactions belonging to this pathway at d7. This temporary shift might be linked to a possible higher hepatic uptake of glutamine, which is used as a co-substrate for some reactions in the pathway, at d7.

Simulations based on the metabolomics data also predicted modulations in the fatty acid oxidation pathway, with smaller fluxes through about 60 fatty acid mitochondrial β-oxidation reactions, which is in line with our previous study showing a reduced expression level of limiting enzymes of this pathway^25^. The reactions predicted to be modulated are specifically those relying on FAD as co-factor. The fact that betaine became significantly taken up at d7 and d60 and that its metabolism also consumes FAD could therefore be linked with the reduction of the fatty acid β-oxidation pathway as a result of FAD competition between reactions.

It should be noted, that one limit of the modelling approach is that the predictions highly depends on the metabolic network model used as a basis: in this study, because there is currently no manually curated, and therefore reliable, reconstruction for the metabolic network of the minipig, or even the pig, we chose to use one of the most up-to-date and consolidated reconstruction of the human metabolic network, Recon2.04. Also, we use the global human metabolic model rather than tissue-specific models since we wanted to be able to compare the analyses made from the metabolomics data obtained both for the liver and intestine. Despite this, it is interesting to notice that the pathways pointed out by the modelling are different from those identified when looking directly at the metabolomics data. By considering the metabolic network and all the interconnections between the metabolic reactions, we are able to predict changes in metabolic pathways that could be the indirect consequence of a differential use of external metabolites. Therefore, we provided strong cues that this modelling approach based on the metabolic network is useful to suggest new and complementary pathways that could not be evidenced by looking directly at the data and would be a helpful tool when digging further into the mechanisms of the observed changes.

### Conclusions and perspectives

Overall, our time-course study of the paired arterial and venous metabolomes across the intestine and liver of HFHS-fed minipigs provided original information about the subtle metabolic regulation taking place during the onset of obesity and insulin resistance^10,25^. Further, our high-throughput approach allowed reflecting the metabolic activity of these organs by providing unique information that could not be obtained by a single venous sampling.

Our approach allowed characterizing the splanchnic area metabolism on healthy minipigs (Fig. 6), including the major role of the liver at the postabsorptive state as supplier of glucose to the peripheral organs, such as the intestine. This glucose output is thought to be fed by different substrates, including muscle-derived lactate and alanine, but also intestinal AA. Finally, we reported that in the absence of luminal nutrients, the intestine was largely taking up glutamine and glutamate, its preferred energy substrates, most likely from muscular and hepatic sources respectively. After two months of HFHS feeding and obesity onset, our study of A/V ratios of the metabolome highlighted three major features: first, the hepatic metabolism shifted from carbohydrate to lipid utilization; second, the energy demand of the intestine increased, resulting in an enhanced uptake of already (d0) used metabolites (glutamine, glutamate), and the recruitment of novel (d7) energy substrates, like choline and creatine; third, other metabolic changes, like the uptake of methionine and threonine were considered to be driven by an increased intestine turnover and epithelium development to cope with a new diet rich in energy and nutrients. Eventually, the unique combination of experimental and modelling-derived data suggested that HFHS feeding would be indirectly associated with modulations of original metabolic pathways, such tryptophan metabolism and fatty acid mitochondrial beta-oxidation, which may play an important role on lipid hepatic accumulation and whole-body insulin sensitivity.

## Supplementary information

Supplementary Figures

Supplementary Dataset

## Data Availability

All data generated or analysed during this study are included in this published article (and its Supplementary Information files). Other datasets generated during and/or analysed during the current study are available from the corresponding author on reasonable request. Metabolomics data will be available on the *MetaboliLights* platform upon acceptance of the article.
