# Supplementary material for: Arterio-venous metabolomics exploration reveals major changes across liver and intestine in the obese Yucatan minipig
Source: Sci Rep. 2019 Aug 29;9:12527. doi: 10.1038/s41598-019-48997-2 (PMC6715693; doi:10.1038/s41598-019-48997-2)

Arterio-venous metabolomics exploration reveals major changes across liver and intestine in the obese Yucatan minipig

Nathalie Poupin[2], Marie Tremblay-Franco[2,4], Aurélien Amiel[2,4], Cécile Canlet[2,4], Didier Rémond[1], Laurent Debrauwer[2,4], Dominique Dardevet[1], Ines Thiele[3,5,6], Maike K. Aurich[3], Fabien Jourdan[2], Isabelle Savary-Auzeloux[1], Sergio Polakof[1*]

Suppl Figure 1: uptake and release profiles of metabolites in intestine and liver

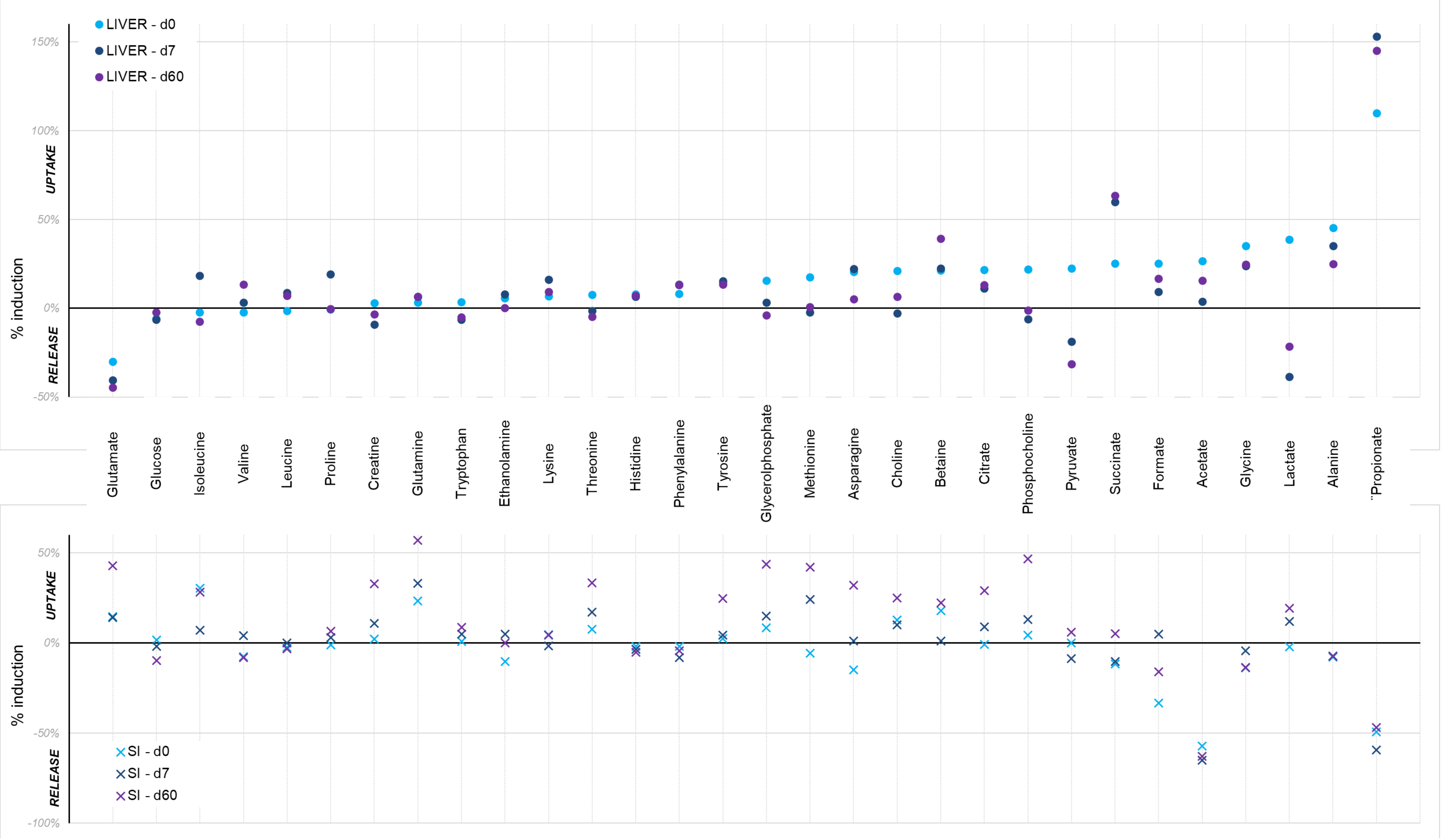

Suppl Figure 2: definition of constraints for exchange reactions based on metabolomics data

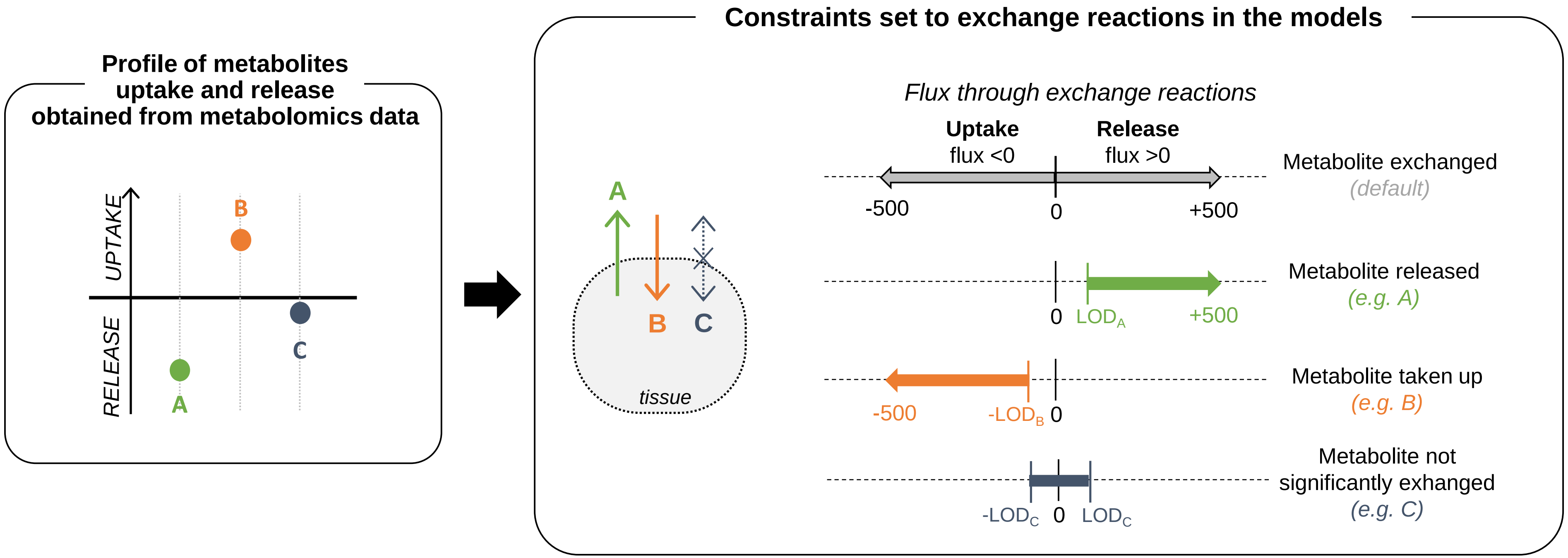

Supplement: Supplementary file 1 — Supplementary Figures [file 41598_2019_48997_MOESM1_ESM.pdf]
